# Clinical and Functional Results Following Posterior Cruciate Ligament Reconstruction in Young Patients

**DOI:** 10.7759/cureus.55058

**Published:** 2024-02-27

**Authors:** Theofylaktos Kyriakidis, Charalampos Pitsilos, Alexandros S Nenopoulos, Vasileios Davitis, Polychronis Papadopoulos, Ioannis Gigis

**Affiliations:** 1 2nd Orthopaedic Department, General Hospital "G. Gennimatas" Aristotle University of Thessaloniki, Thessaloniki, GRC

**Keywords:** hamstring autograft, young patients, clinical evaluation, posterior cruciate ligament injury, posterior cruciate ligament reconstruction (pclr)

## Abstract

Introduction: Posterior cruciate ligament injuries are uncommon, and their management is controversial. However, surgical reconstruction is necessary in case of symptomatic lesions. The present study aimed to analyse patients' reported outcomes and clinical evaluation after isolated posterior cruciate ligament reconstruction.

Materials and methods: The present study includes 12 patients with posterior cruciate ligament rupture. All patients were treated with arthroscopic surgery using single-bundle hamstring autograft ligament reconstruction. The primary outcome was the International Knee Documentation Committee (IKDC) subjective questionnaire; secondary outcomes included the Lysholm score and stability assessment.

Results: At the time of the surgery, the mean age of the study population was 24 years (range: 18-29), with a body mass index (BMI) of 23.2 kg/m^2 ^(range: 21-25), and the mean time from injury was five months (range: 1-8). The follow-up period was at least 24 months. The mean IKDC score significantly increased from 68.0 preoperatively to 92.6 at the final follow-up. The Lysholm score also increased from 68.8 to 95.8. Knee stability was classified as normal in all patients after surgery.

Conclusion: The results of this study indicate that the posterior cruciate ligament reconstruction with single-bundle hamstring autograft is an efficient treatment option for managing symptomatic young patients. All patients presented good functional and clinical results at two years of follow-up. However, further studies with more participants and a longer follow-up are needed to validate these data.

## Introduction

The posterior cruciate ligament (PCL) controls the posterior translation of the tibia on the femur and restricts excessive knee rotation [1]. PCL injuries are relatively rare and result most often after trauma. They are frequently associated with additional knee injuries, including other ipsilateral ligamentous or meniscal pathologies. Their incidence is approximately 3% of all knee injuries and 5-20% of knee ligamentous injuries [2].

In the past, most of these injuries were considered benign and thus treated conservatively with, in fact, satisfactory clinical and functional outcomes [3]. However, the PCL insufficiency that results in knee instability may lead to degenerative changes in the joint, especially in the medial femoral condyle and the patella, and to the development of osteoarthritis, particularly in patients with prior medial meniscectomy [4].

The continuously increasing expectations regarding patients' physical activity and sports, in addition to rigorous daily activities and longer life expectancy, make the surgical management of PCL insufficiency crucial, mainly in the young population. Recent literature has demonstrated that PCL reconstruction is an efficient and safe procedure to cope with knee laxity and decrease functional symptoms and pain [5]. Nevertheless, due to the low incidence of such injuries, limited data exist concerning the clinical and functional outcomes after surgery. Moreover, arthroscopic reconstruction is a challenging and technically demanding procedure and probably is a reason for not performing often [6].

However, the surgical technique for PCL reconstruction is described widely in the literature, and various types of grafts are proposed, with the hamstring still holding a considerable place as a surgeon's choice. Despite the uncertain graft size and the possible muscle weakness, hamstring autografts have essential advantages, as they are easy to harvest and present low donor-site morbidity [7].

The purpose of the present study was to evaluate the outcomes of cruciate ligament reconstruction using single-bundle hamstring autograft in young patients. It was hypothesised that this procedure could provide good clinical and functional outcomes.

## Materials and methods

Between March 2015 and September 2021, 18 patients with PCL rupture were treated surgically using single-bundle hamstring autograft reconstruction. Out of them, six patients presented concomitant ligament injuries and were excluded from further analysis; thus, 12 patients with isolated PCL injuries were included in the current study. Inclusion criteria were female and male subjects with acute PCL injury, under the age of 30, clinical instability and minimum follow-up of 24 months. Exclusion criteria included concomitant ligament injuries, chronic cases more than one year from the injury, inability to follow the rehabilitation program, body mass index over 25 kg/m^2^ and previous knee ligament surgical repair or reconstruction. Demographic characteristics are analytically presented in Table 1.

**Table 1:** Patients' demographic characteristics

Characteristic	Patient data
Sex	Female, n=1
	Male, n=11
Age	24 years (range: 18-29)
Time from injury	5 months (range: 1-8)
Follow-up period	24 months (range: 24-47)
BMI	23.2 kg/m^2^ (range: 21-25)
Side	Right, n=10
	Left, n=2
Mechanism of injury	Sports injuries: 9
	Motor vehicle accident: 3

**Figure 1:**
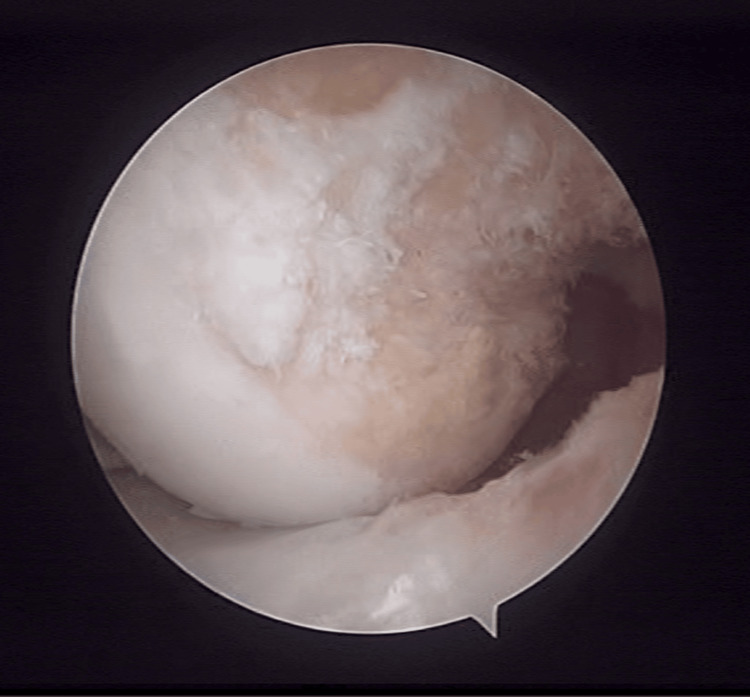
Arthroscopic image of femoral footprint

**Figure 2:**
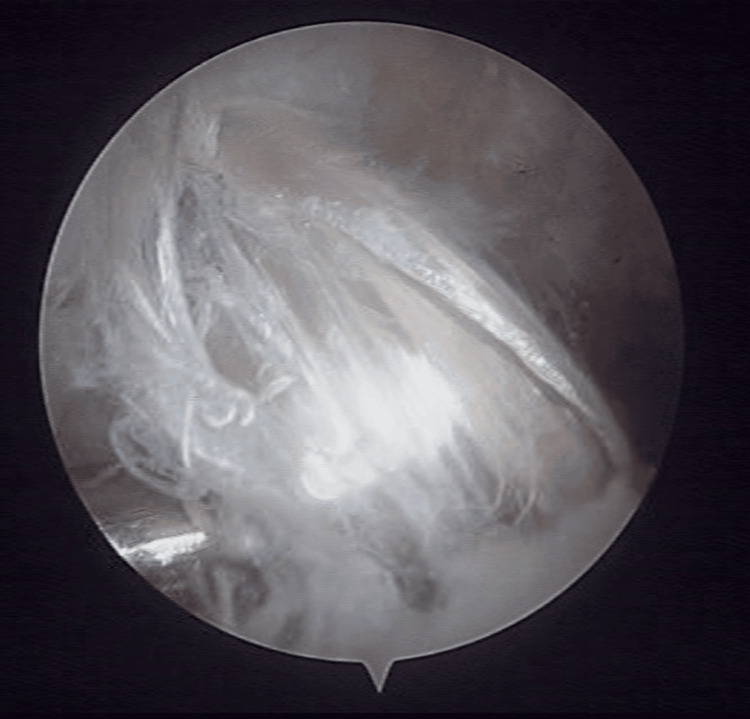
Arthroscopic image of the PCL graft PCL, posterior cruciate ligament.

One senior knee surgeon (IG) with vast surgical experience in sports traumatology performed all surgeries. Patients were placed supine, and the injured knee was placed on a leg holder using an inflated tourniquet at 250 mmHg. An initial arthroscopy was performed for intra-articular evaluation and confirmation of the PCL rupture. After that, the hamstrings were harvested through a longitudinal proximal tibial approach using a blunt tendon stripper. The graft was prepared, and a minimum length of 12 cm was obtained. A cortical adjustable button was then placed on one end and a fixing suture on the other. The procedure was performed through the classic anterolateral (AL) and anteromedial (AM) portals using a 70^ο^ arthroscope. A PCL jig was placed just lateral to the midline of the PCL insertion, and a guide wire was inserted at a 50^ο^ angle. A cannulated reamer was used to create the tibial tunnel with an outside-in method. For the femoral tunnel, a guide wire was passed from the AL portal through the footprint (Figure 1) of the AL bundle of the PCL. A 5 mm cannulated reamer was used to create the tunnel for the cortical button passage through the medial cortex. A cannulated reamer was used to prepare a tunnel of 20 mm depth for graft reception. A guide suture was passed through the femoral tunnel, and the graft was inserted intra-articularly through the AL portal. The cortical button was passed through the femoral tunnel and fixed on the medial cortex. The fixing suture was passed through the tibial tunnel using a suture retriever, and the graft was fixed in the tibia with an interference screw (Figure 2).

The outcomes were assessed using patient-reported outcome measures (PROMs) and clinical evaluation. The primary outcome was the IKDC subjective questionnaire, and secondary outcomes included the Lysholm score and the posterior drawer test classification (Grade I 0-5 mm, Grade II 5-10 mm, and Grade III >10 mm).

Statistical analysis was done by an independent statistician using SPPS 25.0 (IBM Corp, Armonk, NY). Continuous variables were reported as means or medians and categorical values as proportions. All tests were two-sided and statistical significance was assumed for a p-value of <0.05.

## Results

Twelve patients who met the eligibility criteria and were included in the current study were treated with arthroscopic PCL reconstruction using single-bundle hamstring autograft with transtibial technique. The patients were followed for at least 24 months (range 24-47 months). Eleven of them were males and one female, and they had a mean age of 24 years (range: 18-29) and a mean BMI of 23.2 kg/m^2^ (range: 21-25) at the time of surgery. The right leg was involved in 10 cases, while the left was in two cases. The surgery was performed at a mean of five months (range: 1-8) after the injury. Nine cases result from sports accidents, and only three occur after motor vehicle accidents. The mean IKDC score increased significantly from 68 (range: 63.1-76.3) to 92.6 (range: 88.2-97.7) (p<0.05). The Lysholm score improved with a statistically significant difference from 68.8 (range: 63-74) to 95.8 (range: 90-100) (p<0.05). The clinical evaluation using the posterior drawer test demonstrated improvement from Grade III to Grade I in 10 and from Grade III to Grade II in two patients. Detailed analysis of the results is presented in Table 2.

**Table 2:** Detailed analysis of the results IKDC, International Knee Documentation Committee.

Patients	IKDC		Lysholm		Posterior drawer test	
	Preoperative values	Postoperative values	Preoperative values	Postoperative values	Preoperative values	Postoperative values
1	66.4	98.9	74	100	III	I
2	63.1	95.4	73	98	III	I
3	63.2	97.7	70	100	III	I
4	68.8	88.2	58	95	III	II
5	65.6	88.5	66	96	III	I
6	76.3	89.7	63	99	III	I
7	70.1	91.5	74	100	III	I
8	64.8	90.3	73	96	III	I
9	65.2	95.4	69	99	III	I
10	70.1	91.5	67	90	III	II
11	73.1	88.5	69	95	III	I
12	69.0	95.4	70	99	III	I

## Discussion

The most important finding of the study was that PCL reconstruction with transtibial technique using single-bundle hamstring autograft for isolated injury presents satisfactory outcomes regarding both clinical findings and PROMs.

Isolated PCL rupture can be treated conservatively with improved objective and subjective outcomes and a return to the previous activity level [8]. However, PCL deficiency has been correlated with a higher incidence of meniscal tears and symptomatic arthritis in the long term [5]. The strongest indication for PCL reconstruction after isolated injury is persistent symptoms after conservative management [9]. At present, most of the patients report knee instability after at least three months of rehabilitation that adversely affects their daily activities. Only two were operated on less than three months after their injury.

Many different graft types have been used for PCL reconstruction, including autografts and allografts. Regarding autografts, the hamstring tendons have a higher prevalence, followed by bone-patellar tendon-bone, quadriceps tendon, and tibialis anterior tendon [7]. Allografts are more commonly used in combined injuries or revision cases; the Achilles tendon is more frequently applied, while the hamstrings, bone-patellar tendon-bone, and tibialis anterior tendon allografts have also been used [10]. Regarding postoperative outcomes, neither graft has proven clinical or biomechanical superiority [11,12]. The autologous hamstring tendons were used in the present study as they are easily harvested grafts with low donor-site morbidity. All cases were primary, and there were no concomitant ligament injuries.

Three techniques of graft fixation on the tibial side have been described to date: the transtibial, the inlay, and, more recently, the onlay fixation [6,13,14]. While there were concerns about graft elongation due to the "killer turn" in the transtibial technique, the evolution of instrumentation and the research on the optimal angle of the tibial tunnel have reduced the risk of graft failure [15]. In a meta-analysis, Lee et al. [16] found that the transtibial and the tibial inlay fixation are equally efficient in restoring knee stability and function. The onlay technique has also been related to satisfactory outcomes. In this study, we used the transtibial technique using a bioabsorbable interference screw, as this is the preferred fixation method of the surgeon for both isolated and combined PCL injuries. 

The graft used for PCL reconstruction can be placed in a single- or double-bundle fashion. In the former technique, the single bundle mimics the AL bundle of the native PCL. In contrast, in the latter, a second bundle is added to replace the function of the posteromedial PCL bundle [17]. Biomechanical studies suggest that the single-bundle technique is inferior to the double-femoral bundle and may be associated with increased postoperative knee laxity [18]. However, in clinical practice, PCL reconstruction in single- or double-bundle fashion has no difference in the functional outcome [19]. Krott et al. [20], in a meta-analysis of 13 studies including 603 patients, compared the single- and double-bundle PCL reconstruction and concluded that both techniques were associated with similar satisfactory outcomes concerning motion, stability, and overall function. The present study uses the single-bundle technique with cortical button fixation at the femur with satisfactory functional outcomes. 

The sufficient restoration of knee kinematics after PCL reconstruction remains uncertain [21]. Jenner et al. [22] found that 28% of 18 patients had abnormal knee stability at 3.3 years of follow-up after PCL reconstruction for chronic instability. On the other hand, in a more recent study, Boutefnouchet et al. [23] reported that only one out of 15 patients (7%) did not yield normal or nearly normal knee kinematics using the IKDC evaluation form after transtibial PCL reconstruction with hamstring autograft at 4.1 years. Studying the efficacy of the same technique in 23 patients with isolated PCL injury, Ochiai et al. [24] found at two years an improvement of the tibial translation ratio on lateral stress X-rays from 42% to 51.5% and a reduction of side-to-side difference in tibial translation from 8.9 mm to 4.2 mm. The present study evaluated the posterior drawer test, and all patients improved from type III to type II or I.

PCL reconstruction after isolated injury using hamstring tendons autograft with transtibial technique has been related to satisfactory mid- to long-term PROMs and daily activity level. Lahner et al. [25] found an increase in the IKDC score from 41.9 to 69.5 and the Tegner activity score from 2.8 to 5.9 points at a two-year follow-up of 33 patients after isolated PCL reconstruction. Of them, 24 patients (72.8%) reported that their knee function was normal or nearly normal. In a similar study, Norbakhsh et al. [26] evaluated 52 patients and found an improvement in Lysholm score from 59 to 90 at a minimum of three years of follow-up. Forty-one out of 52 included patients (79%) exhibited normal or nearly normal knee function. In a mean of 27.1 months after PCL reconstruction, Eguchi et al. [27] found that the Lysholm score of the 19 included patients significantly increased from 63.7 to 94.4. Studying 12 patients, Ihle et al. [28] found improvement in the Hospital for Special Surgery score from 74.3 to 88.3 and in Lysholm score from 46.4 to 84.7 at a mean period of 51 months after PCL reconstruction.

Additionally, they found a mean IKDC score of 80 and a Tegner activity scale of 4.8 at the final evaluation without reporting the pre-operative status. Finally, they found that 83% could exercise their occupation with the same physical workload as before the accident. In this study, both the IKDC and the Lysholm scores were improved in the final follow-up. The IKDC score increased from 68 to 92.6, and the Lysholm score rose from 68.8 to 95.8. 

This study also has some limitations. First, the patient sample was relatively small. However, the isolated symptomatic PCL rupture may be treated conservatively with satisfactory outcomes, and the cases refractory to rehabilitation are relatively rare. Second, there was a short- to mid-term follow-up period, so we could not assess the prevalence of related degenerative osteoarthritis. However, all the operations were performed by a single senior surgeon.

## Conclusions

The results of this study indicate that the PCL reconstruction with single-bundle hamstring autograft is an efficient treatment option for managing symptomatic young patients. All patients presented good functional and clinical results at two years of follow-up. However, further studies with more participants and a longer follow-up are needed to validate these data.
